# Successful Non-Surgical Deep Uterine Transfer of Porcine Morulae after 24 Hour Culture in a Chemically Defined Medium

**DOI:** 10.1371/journal.pone.0104696

**Published:** 2014-08-13

**Authors:** Emilio A. Martinez, Miguel Angel Angel, Cristina Cuello, Jonatan Sanchez-Osorio, Jesus Gomis, Inmaculada Parrilla, Jordi Vila, Ignaci Colina, Marta Diaz, Josep Reixach, Jose Luis Vazquez, Juan Maria Vazquez, Jordi Roca, Maria Antonia Gil

**Affiliations:** 1 Department of Animal Medicine and Surgery, University of Murcia, Murcia, Spain; 2 Department of Research and Development, Selección Batallé S.A., Girona, Spain; Rutgers University -New Jersey Medical School, United States of America

## Abstract

Excellent fertility and prolificacy have been reported after non-surgical deep uterine transfers of fresh in vivo-derived porcine embryos. Unfortunately, when this technology is used with vitrified embryos, the reproductive performance of recipients is low. For this reason and because the embryos must be stored until they are transferred to the recipient farms, we evaluated the potential application of non-surgical deep uterine transfers with in vivo-derived morulae cultured for 24 h in liquid stage. In Experiment 1, two temperatures (25°C and 37°C) and two media (one fully defined and one semi-defined) were assessed. Morulae cultured in culture medium supplemented with bovine serum albumin and fetal calf serum at 38.5°C in 5% CO_2_ in air were used as controls. Irrespective of medium, the embryo viability after 24 h of culture was negatively affected (P<0.05) at 25°C but not at 37°C compared with the controls. Embryo development was delayed in all experimental groups compared with the control group (P<0.001). Most of the embryos (95.7%) cultured at 37°C achieved the full or expanded blastocyst stage, and unlike the controls, none of them hatched at the end of culture. In Experiment 2, 785 morulae were cultured in the defined medium at 37°C for 24 h, and the resulting blastocysts were transferred to the recipients (n = 24). Uncultured embryos collected at the blastocyst stage (n = 750) were directly transferred to the recipients and used as controls (n = 25). No differences in farrowing rates (91.7% and 92.0%) or litter sizes (9.0±0.6 and 9.4±0.8) were observed between the groups. This study demonstrated, for the first time, that high reproductive performance can be achieved after non-surgical deep uterine transfers with short-term cultured morulae in a defined medium, which opens new possibilities for the sanitary, safe national and international trade of porcine embryos and the commercial use of embryo transfer in pigs.

## Introduction

It is recognized that embryo transfer (ET) has the potential to greatly benefit the pig industry because it allows the movement of genetic resources with minimal risk of disease transmission, reduced transportation costs and no impact on animal welfare during transport. Despite these extraordinary advantages, the commercial utilization of ET in pigs, unlike with other livestock, remains very limited due to the lack of effective non-surgical ET instruments. The cervical folds and the length and coiled nature of the pig uterine horns have been the principal obstacles for the development of an effective procedure for non-surgical ET over the past decades [1]. In the 1990s, several non-surgical procedures to deposit morulae and/or blastocysts into the cervix or uterine body were developed [2], [3], but the farrowing rates (<40%) and litter sizes (5–7.5 piglets born) were lower compared with those previously reported using surgical ET (farrowing rates: 80%; litter sizes: 8 piglets born) [4]. Because the middle and last third of the uterine horn are more physiologically appropriate locations for these embryos than the cervix or the uterine body, we developed a new and unique procedure for non-surgical deep uterine (NsDU) transfer of porcine embryos with acceptable and promising reproductive performance of the recipients (71.4% farrowing rate and 6.9 piglets born) [5]. With the recent improvement of the procedure, the results have been greatly increased (85% farrowing rate and 9.8 piglets born) [6], opening new possibilities for the commercial use of ET technology in the pig industry.

All the studies mentioned above used surgical or non-surgical transfer of fresh embryos, which were transferred immediately after collection or a few hours later. However, from a commercial point of view, the embryos must be stored until they are transferred to the recipient farm, which implies the need for their national or international transport. Vitrification is the only suitable method for the long-term storage of porcine embryos. With current methods, high percentages of morulae and blastocysts survive the vitrification and warming procedures, and high farrowing rates (75%) and litter sizes (10 piglets born) have been obtained with these embryos after surgical transfer to recipients. Unfortunately, when embryo vitrification and NsDU-ET are combined, a severe decrease in the reproductive performance of the recipients has been noted (reviewed in [7]). Although several research programs are in progress to improve these results, an alternative method to maintain the developmental capacity of the embryos from collection until transfer is in vitro culture, which can be used as a method for medium-term (3–4 days) or short-term (24 h) embryo storage. Because transportation is restricted to embryos with an intact zona pellucida (ZP) for hygienic reasons, the most appropriate stages for commercial ET are the morula and unhatched blastocyst. For medium-term storage, the embryos must be collected at a very early developmental stage, which prevents development beyond the unhatched blastocyst stage at the end of the culture. Although high blastocyst formation rates from one-, two- and four-cell embryos cultured in vitro for 3–4 days has been reported [8], [9], these blastocysts had a lower total cell number [9], [10] and in vivo developmental potential [11] compared with their in vivo counterparts. The harmful effects of the culture conditions on embryo development might be minimized by using a shorter period of culture, such as 24 h. This period will allow for the short- and medium-distance transportation of the embryos from the donor to the recipient farm. In this case, the embryos should be collected at the morula or early blastocyst stages, which should not develop beyond the unhatched blastocyst stage during the 24 h of culture. Despite its importance, research on short-term porcine embryo culture has been very limited. Nonetheless, some promising findings have been reported, including the acceptable farrowing rates (50%) and litter sizes (6 piglets born) obtained after surgical transfers of embryos cultured in serum-containing medium for 30 h at 36.5°C [12], which indicates that short-term cultured embryos are able to develop to term. In addition, several types of serum-containing or bovine serum albumin (BSA)-containing media and several temperatures have been shown to be effective for short-term embryo culture [13], although the in vivo developmental capacity after the transfer of the cultured embryos was not evaluated in that study. Unfortunately, to the best of our knowledge, there are no reports in the literature that address an important aspect for the practical use of short-term embryo culture: the potential use of chemically defined culture media to allow safe embryo transportation and transfer. Generally, the media intended for embryo culture are supplemented with either serum or serum components, which carries a risk of disease transmission [14], [15], and this is an important limitation for embryo movement. Similarly, there has been no research on the use of short-term cultured embryos in combination with NsDU-ETs, which is fundamental for practical ET.

This study aimed to evaluate the potential application of NsDU-ET with in vivo-derived morulae cultured for 24 h. We determined (1) the effects of culture temperature (25°C and 37°C) on the in vitro viability and development of embryos cultured in semi-defined and defined media and (2) the reproductive performance of recipients after NsDU transfer of embryos cultured at 37°C in a defined medium.

## Materials and Methods

### Chemicals

All chemicals used in this study were purchased from Sigma-Aldrich Quimica SA (Madrid, Spain) unless otherwise noted.

### Animals

Weaned purebred Duroc sows (2–7 parity) were used as donors and recipients. Females were allocated individually to crates in a mechanically ventilated confinement facility at a pig genetics company (Selección Batallé S.A., Girona, Spain). The animals were fed a commercial ration twice per day, and water was provided ad libitum.

All experimental procedures used in this study were performed in accordance with the 2010/63/EU EEC Directive for animal experiments and were reviewed and approved by the Ethical Committee for Experimentation with Animals of the University of Murcia, Spain (research code: 3001/2009).

### Superovulation and detection of estrus

Weaning was used to synchronize the estrus in donors and recipients. To standardize the ET schedule, only sows with a weaning-to-estrus interval of 3–4 days were selected as donors and recipients. The superovulation of donors was induced by the intramuscular administration of 1000 IU equine chorionic gonadotropin (eCG; Foligon, Intervet, Boxmeer, The Netherlands) 24 h after weaning. Estrus was checked twice per day by exposing sows to a mature boar (nose-to-nose contact) and applying manual back pressure. Females that exhibited a standing estrous reflex were considered to be in estrus. Only sows with clear signs of estrus at 48–72 h post-eCG administration were further intramuscularly administered with 750 IU of human chorionic gonadotropin (Veterin Corion, Divasa, Farmavic S.A., Barcelona, Spain) at the onset of estrus.

### Insemination of donors

Rich fractions of ejaculates were manually collected from healthy, sexually mature Duroc boars (2–3 years of age) that were fertile and undergoing regular semen collection for commercial artificial insemination. Post-cervical inseminations were performed at 0 h, 24 h and 36 h after the onset of estrus. Insemination doses (1.5 × 10^9^ spermatozoa in 45 mL) were prepared from semen diluted in Beltsville thawing solution (BTS) extender [16] and stored for a maximum of 72 h at 18°C.

### Embryo recovery and evaluation

Embryo collection was performed in a surgical room located on the farm. The donors were subjected to a midventral laparotomy on Days 5 and 6 of the estrous cycle (Day 0: onset of estrus) to obtain morulae and unhatched blastocysts, respectively. The donors were sedated by the administration of azaperone (2 mg/kg body weight, intramuscular). General anesthesia was induced using sodium thiopental (7 mg/kg body weight, intravenous) and maintained with isoflurane (3.5–5%). After exposure of the genital tract, the corpora lutea on the ovaries were counted. Embryos were collected by flushing the tip of each uterine horn with 30 mL of a chemically defined medium consisting of Tyrode's lactate (TL)-HEPES-polyvinyl alcohol (PVA) [17] with some modifications. This medium (TL-PVA) was composed of 124.3 mM NaCl, 3.2 mM KCl, 2 mM NaHCO_3_, 0.34 mM KH_2_PO_4_, 10 mM Na-lactate, 0.5 mM MgCl_2_·6H_2_O, 2 mM CaCl_2_·2H_2_O, 10 mM HEPES, 0.2 mM Na-pyruvate, 12 mM sorbitol, 0.1% (w/v) PVA, 75 µg/ml potassium penicillin G and 50 µg/mL streptomycin sulfate. The collected embryos were evaluated to verify their developmental stage and quality grade. One-cell eggs and poorly developed embryos were classified as oocytes and degenerate embryos, respectively. The remaining embryos that exhibited appropriate morphology according to the criteria determined by the International Embryo Transfer Society [18] were considered viable. Only compacted morulae and unhatched blastocysts graded as excellent or good based on morphological appearance were used in the experiments according to the specific experimental design.

The ovulatory response of the donors was determined by counting the number of corpora lutea in both ovaries. To evaluate the effectiveness of the superovulation treatment, the numbers of viable embryos and oocytes and degenerate embryos were counted in each donor. The recovery rate was defined as the ratio of the number of embryos and oocytes and degenerate embryos recovered to the number of corpora lutea present. The fertilization rate was defined as the ratio of the number of viable embryos to the total number of embryos and oocytes and degenerate embryos collected.

### Embryo culture and assessment of embryo development stage and total cell number

The collected embryos were washed ten times in TL-PVA medium and cultured for 24 h in different media and at different temperatures according to the experimental design. At 0 h and 24 h of culture, all embryos were photographed and retrospectively analyzed to determine their stage of development and quality. The developmental stage was considered linear for the purposes of statistical analysis and scored as previously described [10] according to the following classes: 1, morula; 2, early blastocyst (blastocyst with an incipient visible blastocoele); 3, full blastocyst (blastocyst with a well-defined blastocoele, inner cell mass and trophoblast totally discernible); 4, expanded blastocyst (blastocyst with overall diameter increased and ZP tinned); and 5, hatching or hatched blastocyst (blastocyst with broken ZP or without ZP). The outer diameter (including the ZP) and the thickness of the ZP of each embryo were measured using the open-source software ImageJ (National Institutes of Health; http://rsbweb.nih.gov/ij/). For evaluation of total cell number, embryos were fixed in 4% paraformaldehyde in phosphate-buffered saline (PBS) for 30 min at room temperature (22–24°C), washed twice with PBS containing 3 mg/mL BSA, mounted on a slide in 4 µL of Vectashield (Vector, Burlingame, CA, USA) containing 10 µg/mL Hoechst 33342 and covered with a coverslip. Fixed embryos were examined with fluorescence microscopy using an excitation filter of 330–380 nm. The total number of nuclei that displayed blue fluorescence was counted. The same person performed all cell counts.

### Non-surgical deep uterine embryo transfer

The NsDU-ETs were conducted in non-hormonally treated recipient sows using a previously described method [6]. Briefly, six hours prior to transfer, each recipient received a single intramuscular injection of a long-acting amoxicillin suspension (Clamoxyl LA; Pfizer, Madrid, Spain) at a dosage of 15 mg/kg. Recipients were housed in gestation crates in a small room (12 crates) exclusively used for that purpose. The perineal area of the recipients was thoroughly cleaned, and the vulva was then washed and decontaminated with chlorhexidine. Non-surgical ET catheters (Deep Blue ET catheter, Minitüb, Tiefenbach, Germany) were used for the transfers. When the catheter was completely inserted into one uterine horn, a 1 mL syringe containing the embryos in 0.1 mL of TL-PVA medium was connected to the catheter, and the contents were introduced into the catheter. An additional volume of 0.3 mL of TL-PVA medium was used to force the embryos out of the catheter into the uterus.

### Experimental design

#### Experiment 1

A 2×2 factorial design experiment in two replicates was conducted to evaluate the influence of culture temperature and medium on in vitro development of morulae cultured for 24 h. A total of 12 donors were selected based on their reproductive history (farrowing rate: 90.0%, litter size: 10.3 ± 0.4 piglets, parity number: 3.0 ± 0.2, and lactation length: 22.8 ± 0.5 days). Embryo collection was performed on day 5 of the cycle to collect morulae. Immediately after collection, groups of 7–10 morulae were cultured for 24 h at 25°C or 37°C in Eppendorf tubes containing 1.5 mL of chemically defined TL-PVA medium or NCSU-23 medium [19] supplemented with 10 mM HEPES and 0.4% BSA (semi-defined medium; NCSU-BSA). Morulae cultured (7–10 per well) in a 4–well multi–dish plate containing 500 µL of NCSU-23 medium supplemented with 0.4% BSA and 10% fetal calf serum at 38.5°C in humidified air with 5% CO2 were used as controls. Embryos from each donor were equally and randomly allocated to each of the groups. The embryos were evaluated for developmental progression to the blastocyst stage and total blastocyst cell number as described above. Morulae that progressed during culture to the blastocyst stage were considered viable. The in vitro survival rate was defined as the ratio of viable embryos divided by the total number of cultured embryos. The hatching rate was defined as the ratio of the number of hatching or hatched embryos to the total number of cultured embryos.

#### Experiment 2

This experiment was performed to evaluate the in vivo development of blastocysts derived from cultured morulae. A total of 77 donors were selected based on their reproductive history (farrowing rate: 91.0%, litter size: 11.5 ± 0.2 piglets, parity number: 4.9 ± 0.2, and lactation length: 23.2 ± 0.2 days). Donors were subjected to laparotomy on Days 5 and 6 of the cycle to collect morulae and blastocysts, respectively. Embryos at the morulae stage were cultured in TL-PVA medium at 37°C for 24 h as described above. At the end of the culture, the embryos were morphologically evaluated, and those progressing to the blastocyst stage were transferred to the recipients. A control group was established in which uncultured embryos collected at the blastocyst stage were directly transferred to the recipients within 3 h of collection.

All transfers to recipients were conducted on Day 5 of the estrous cycle. The recipients (n = 49) were selected based on their reproductive history and body condition. There were no differences in the reproductive history of the recipients assigned to each of the two groups (farrowing rates: 85.7% and 92.3%; litter sizes: 11.4 ± 0.3 and 11.6 ± 0.3 piglets; parity number: 5.2 ± 0.2 and 5.3 ± 0.2; lactation length: 25.2 ± 0.4 and 25.7 ± 0.5 days). Thirty transferable un-hatched blastocysts were non-surgically transferred to the depth of a uterine horn of each recipient. The same operator performed all transfers. The experiment comprised a total of 5 trials. Each trial was conducted in separate sessions over a 1–year period and consisted of 14–16 donors and 9–11 transfers. Within each trial, the donors were inseminated with sperm doses from the same boar, and a similar number of recipients with transferred embryos from the two groups (cultured and uncultured blastocysts) was included. Starting 12 days after NsDU-ETs, the recipients were checked daily for signs of estrus. Pregnancy was diagnosed by ultrasonography on day 20 post–transfer. All pregnant sows were allowed to carry litters to term, and farrowing rates and litter sizes were recorded. The in vivo embryo survival rate was calculated as the ratio of the number of piglets born alive to the number of embryos transferred to all recipients.

### Statistical analysis

The data were analyzed using the IBM SPSS 19 Statistics package (SPSS, Chicago, IL, USA). The percentage data were compared using Fisher's exact test. The development stage scores were analyzed using Kruskal–Wallis' test. As this test revealed significant differences, two-by-two comparisons were made using a Mann–Whitney U-test for two independent samples. Continuous variables were evaluated using the Kolmogorov–Smirnov test to assess the assumption of normality, and groups were compared by mixed-model ANOVA (Experiment 1) or Student's t-test (Experiment 2). The ANOVA model included the fixed effects of the temperature, the medium and their interaction, and the random effect of the replicate. When the ANOVA revealed a significant effect, values were compared using Bonferroni's test. The coefficient of variation (CV, standard deviation/mean) was used as a measure of variability of the ovulatory response. Differences were considered significant when p<0.05. The results are expressed as percentages and means ± S.E.M.

## Results

### Experiment 1

Of 12 donors, 8 (66.7%) and 2 (16.7%) sows had embryos at the morula and blastocyst stage, respectively, whereas the remaining two donors had oocytes after flushing. The mean ovulation rate in pregnant sows was 25.2 ± 1.5 corpora lutea (range 19 to 31 corpora lutea, CV  = 17.3%). The recovery and fertilization rates were 96.5% and 95.5%, respectively, and the mean number of viable embryos and oocytes and/or degenerate embryos obtained in the pregnant sows was 23.2 ± 1.6 and 1.1 ± 0.2, respectively. Only embryos at the morula stage (n = 212) were used in this experiment. The embryos collected at the blastocyst stage were used for other studies.

**Figure 1:**
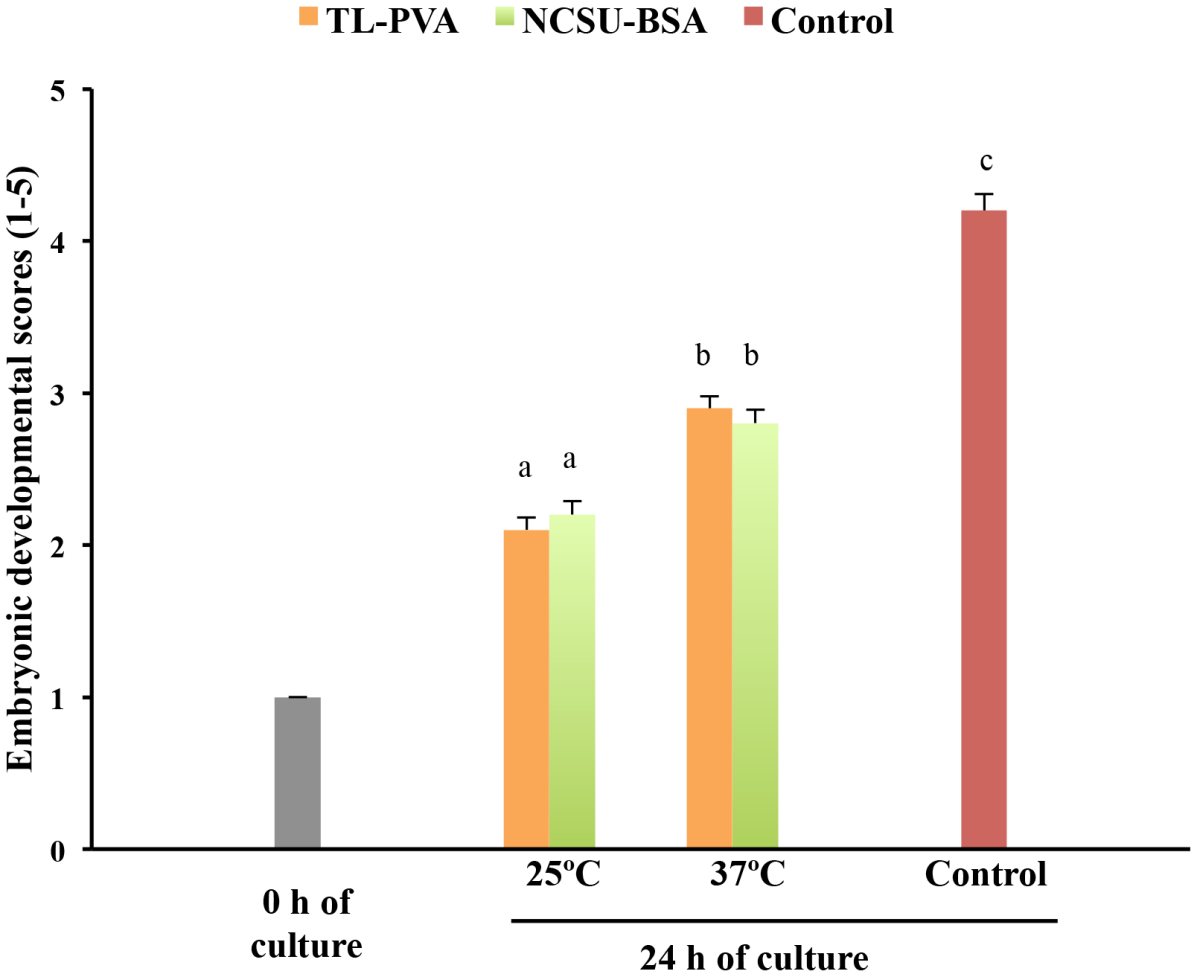
Developmental scores (means ± S.E.M.) of in vivo-derived morulae cultured for 24 h using different temperatures and media. Morulae were cultured in closed Eppendorf tubes containing 1.5 mL of TL-PVA or NCSU23-BSA media, supplemented with 10 mM HEPES, at 25°C or 37°C. Controls were morulae cultured in 4-well multi-dish plates, with each well containing 500 µL of NCSU-23 medium supplemented with BSA and fetal calf serum at 38.5°C in humidified air with 5% CO_2_. The developmental stage was scored according to the following classes: 1, morula; 2, early blastocyst; 3, full blastocyst; 4, expanded blastocyst; and 5, hatching or hatched blastocyst. Different letters represent differences (p<0.001) in developmental scores among groups.

**Figure 2:**
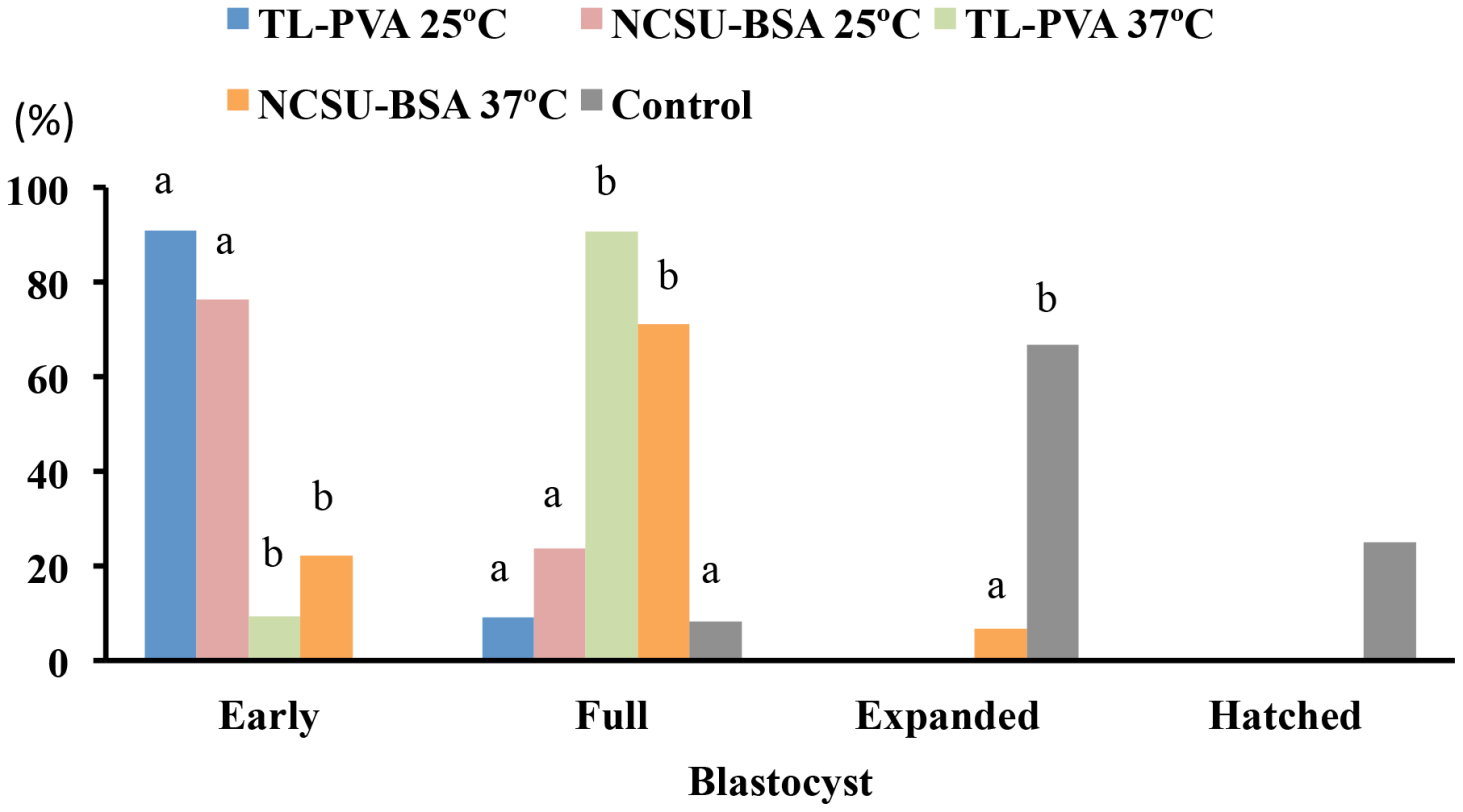
Frequency distribution of in vivo-derived morulae cultured for 24 h using different temperatures and media. Morulae were cultured in closed Eppendorf tubes containing 1.5 mL of TL-PVA or NCSU23-BSA media, supplemented with 10 mM HEPES, at 25°C or 37°C. Controls were morulae cultured in 4-well multi-dish plates, with each well containing 500 µL of NCSU-23 medium supplemented with BSA and fetal calf serum, at 38.5°C in humidified air with 5% CO_2_. Different letters within each blastocyst stage represent differences (p<0.001) in developmental frequencies among groups.

**Figure 3:**
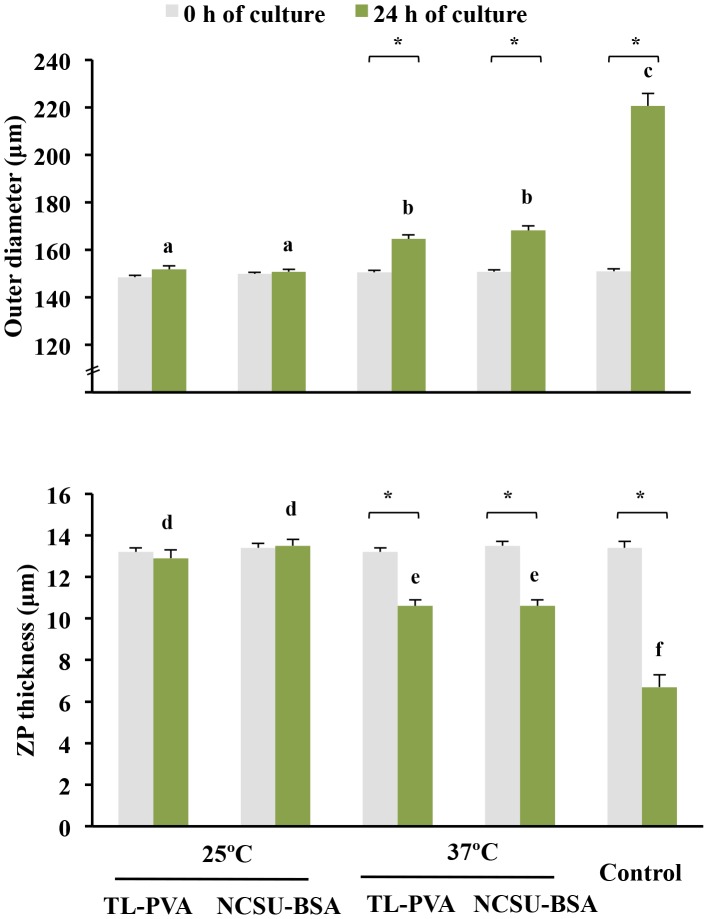
Outer diameter and zona pellucida (ZP) thickness (means ± S.E.M.) of in vivo-derived morulae cultured for 24 h using different temperatures and media. Morulae were cultured in closed Eppendorf tubes containing 1.5 mL of TL-PVA or NCSU23-BSA media, supplemented with 10 mM HEPES, at 25°C or 37°C. Controls were morulae cultured in 4-well multi-dish plates, with each well containing 500 µL of NCSU-23 medium supplemented with BSA and fetal calf serum, at 38.5°C in humidified air with 5% CO_2_. Different letters represent differences (a, b, c: p<0.002; d, e, f: p<0.02) in embryo diameter and ZP among groups. Asterisks indicate differences (P<0.001) between 0 h and 24 h of culture within each group.

**Figure 4:**
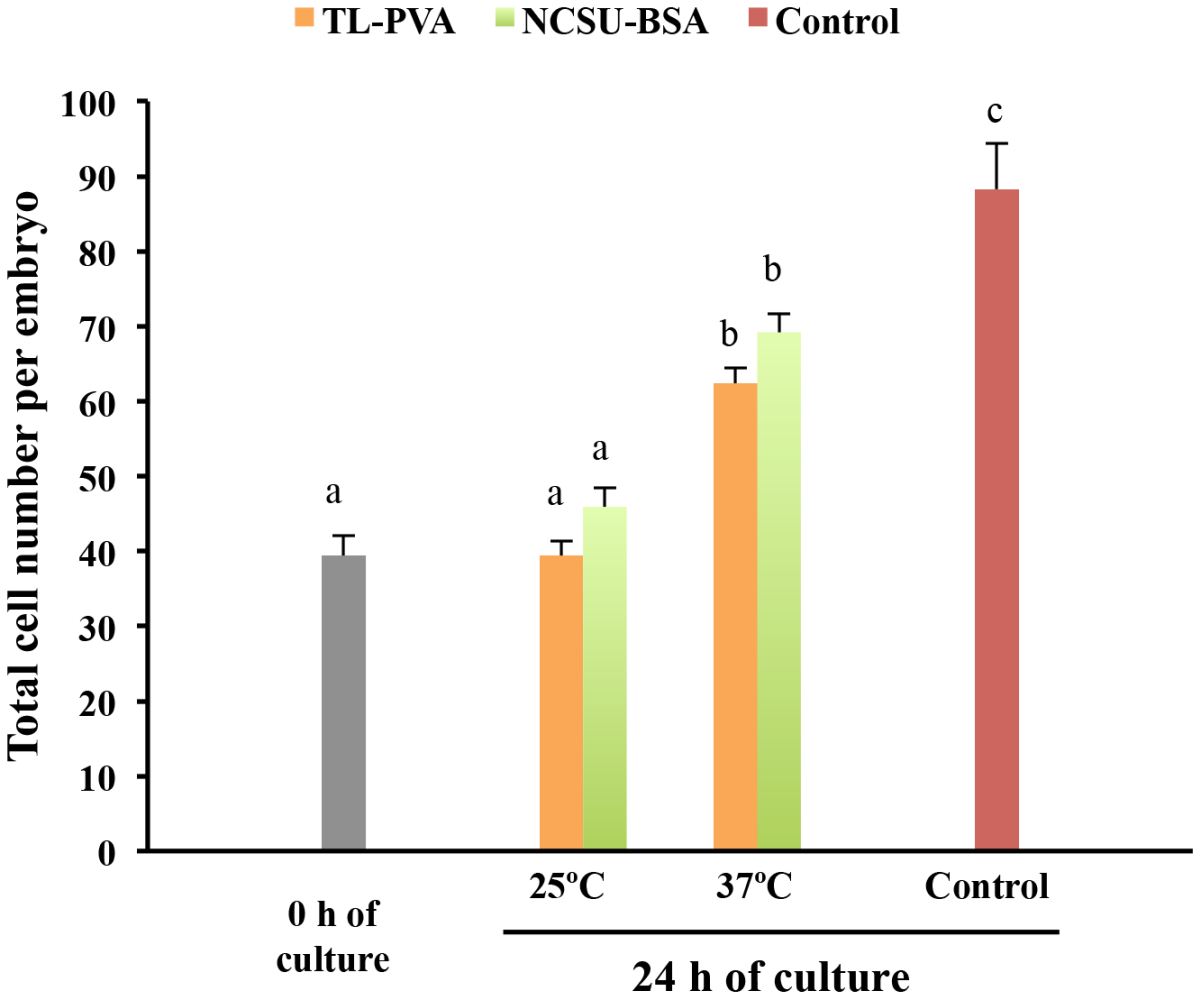
Total cell number (means ± S.E.M.) of in vivo-derived morulae cultured for 24 h under different temperatures and media. Morulae were cultured in closed Eppendorf tubes containing 1.5 mL of TL-PVA or NCSU23-BSA media, supplemented with 10 mM HEPES, at 25°C or 37°C. Controls were morulae cultured in 4-well multi-dish plates, with each well containing 500 µL of NCSU-23 medium supplemented with BSA and fetal calf serum, at 38.5°C in humidified air with 5% CO_2_.Different letters represent differences (p<0.001) among groups.

**Figure 5:**
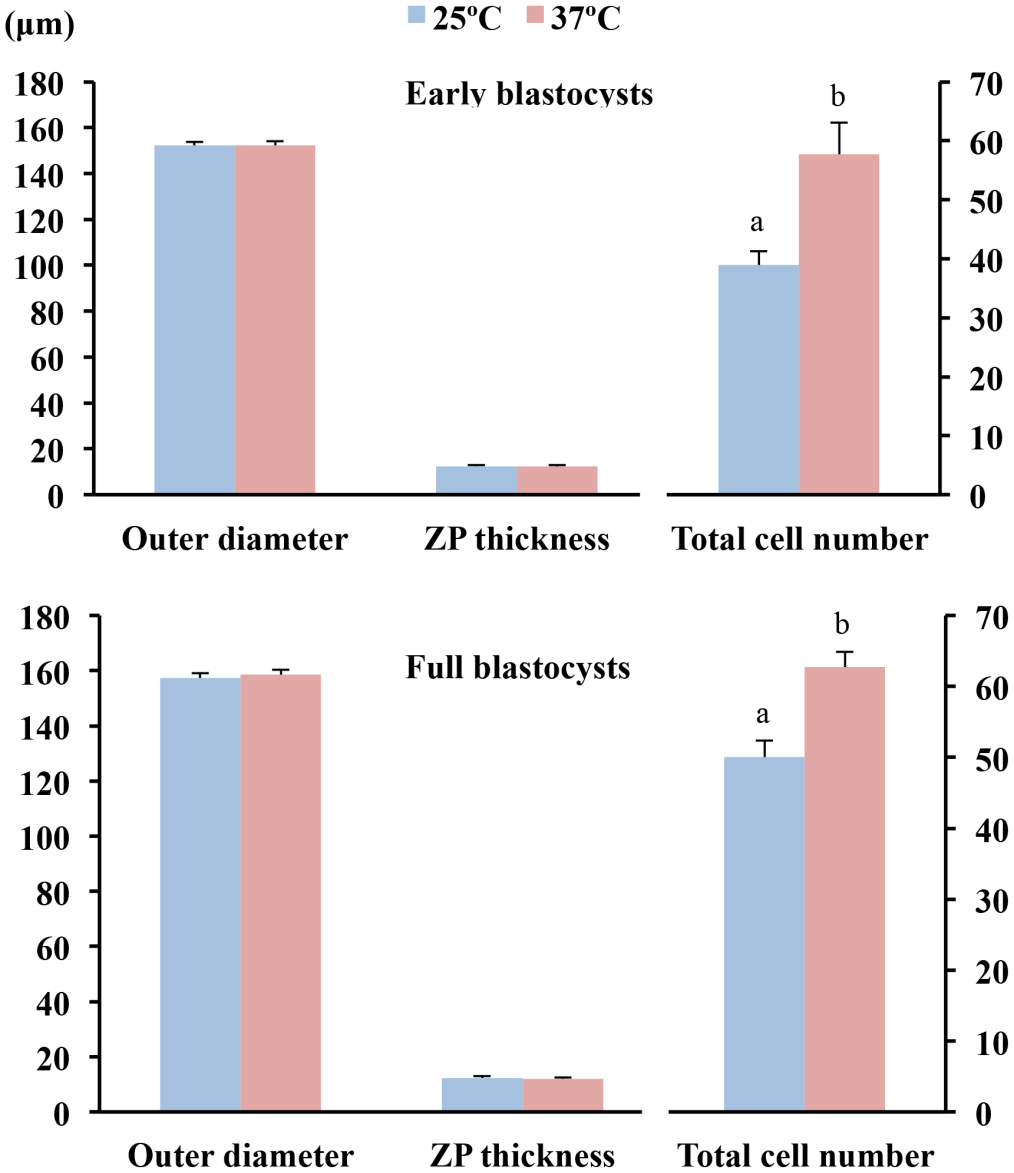
Cell numbers of blastocysts with similar sizes derived from morulae cultured for 24 h at 25°C or 37°C. The total cell count (means ± S.E.M.) was performed in selected embryos in the early blastocyst (n = 30) and the full blastocyst (n = 24) stages. Different letters represent differences (P<0.001) in the total cell number between groups.

While the control embryos had a 100% survival rate after 24 h of culture, a decrease (p<0.05) in viability was observed for embryos cultured at 25°C, regardless of the medium used. Embryos cultured at 37°C in TL-PVA or NCSU-BSA media exhibited survival rates above 95%, with no differences compared with the control group (Table 1). As shown in Figure 1, embryo development in all experimental groups was delayed at 24 h of culture compared with that of the control group (p<0.001). This delay was more profound (p<0.001) when the embryos were incubated at 25°C, regardless of the medium selected. At the end of the culture, 80.0% of the embryos cultured at 25°C achieved the early blastocyst stage, whereas 82.0% of the embryos cultured at 37°C achieved the full or expanded blastocyst stages. Unlike the controls, none of the embryos cultured in the experimental groups hatched at the end of the culture period (Figure 2). At 24 h of culture and corresponding with these data, the outer diameter and the number of cells of the embryos in the experimental groups increased (p<0.002) and the ZP thickness decreased (p<0.02) with increasing culture temperature, regardless of the medium. There was no temperature × medium interaction. The control embryos had a larger (p<0.001) diameter, more cells, and a smaller (p<0.001) ZP thickness than those observed in the experimental groups. There were differences (p<0.001) in the diameter, ZP thickness and number of cells between 0 h and 24 h of culture for the 37°C and control groups but not for embryos cultured at 25°C (Figures 3 and 4). When embryos at the early blastocyst or full blastocyst stages with similar outer diameters and ZP thicknesses were selected, differences (P<0.004) in the total cell number between the embryos cultured at 25°C and 37°C were also evident (Figure 5).

**Table 1:** In vitro survival of in vivo-derived morulae cultured for 24 h using different temperatures and media.

Temperature (°C)	Media#	No.	No. (%) of viable embryos
25	TL-PVA	48	22 (45.8)^a^
	NCSU-BSA	46	38 (82.6)^b^
37	TL-PVA	47	45 (95.7)^bc^
	NCSU-BSA	47	45 (95.7)^bc^
			
38.5	Control	24	24 (100.0)^c^

# TL-PVA and NCSU-BSA media were supplemented with 10 mM HEPES. Control: embryos were cultured in NCSU-23 medium supplemented with 0.4% BSA and 10% fetal calf serum at 38.5°C in humidified air with 5% CO2.

a,b,c Values with different letters differ by P<0.05.

### Experiment 2

**Figure 6:**
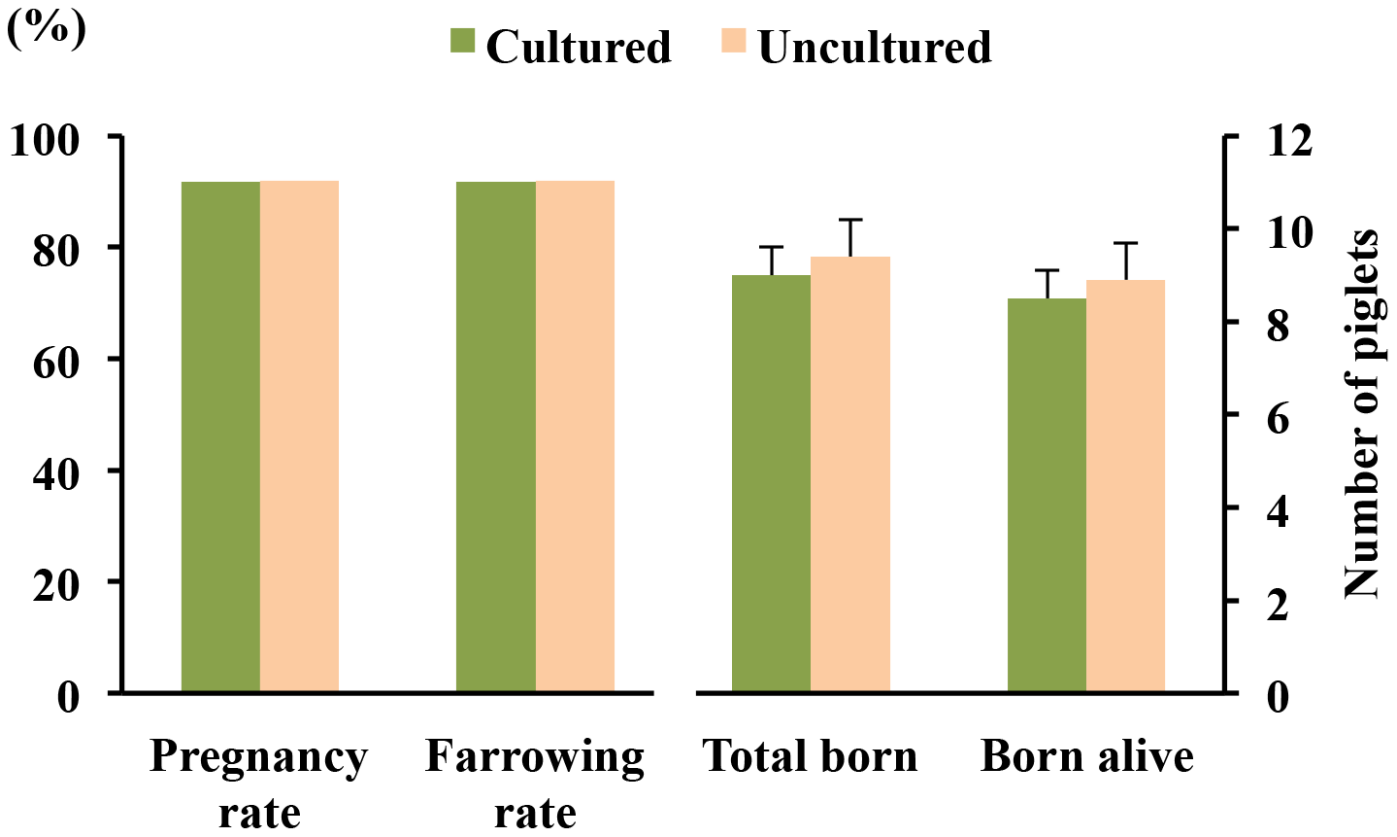
Reproductive performance of recipients after non-surgical deep uterine embryo transfers. Fertility (%) and prolificacy (mean ± S.E.M.) following transfer of blastocysts derived from morulae cultured for 24 h at 37°C in TL-PVA supplemented with 10 mM HEPES (n = 24). Uncultured embryos collected at the blastocyst stage were directly transferred to the recipients within 3 h of collection (n = 25).

Of 77 donors, 74 (96.1%) had morulae and blastocysts on Days 5–6 of the cycle, and 3 (3.9%) had only oocytes. The mean ovulation rate was 23.9 ± 0.5 corpora lutea (range 16 to 40 corpora lutea, CV  = 17.7%). The recovery and fertilization rates were 95.4% and 94.3%, respectively, and the mean number of viable embryos and oocytes and/or degenerate embryos per pregnant sows was 21.5 ± 0.5 and 1.3 ± 0.3, respectively. A total of 737 out of 785 (93.9%) morulae were viable at 24 h of culture, achieving the early (23.9%), full (67.1%) or expanded (9.0%) blastocyst stages. Blastocysts derived from culture (n = 720) and uncultured embryos collected at the blastocyst stage (n = 750) were transferred to a total of 24 and 25 recipients, respectively. The reproductive performance of recipients after transfers is presented in Figure 6. No differences in farrowing rates (91.7% and 92.0%) or litter sizes (9.0±0.6 and 9.4±0.8) were observed between the groups (cultured and uncultured, respectively). Additionally, there were no differences between groups with respect to piglet birth weight (1.6±0.1 and 1.5±0.1 kg), embryo survival rates (27.5% and 28.8%) or sex ratio at birth (male/female: 55.6/44.4 and 53.1/46.9).

## Discussion

This is the first study to demonstrate that porcine morulae can be successfully cultured short-term for up to 24 h at 37°C in a chemically defined medium and that the in vivo ability to develop to term after NsDU-ET of the resulting blastocysts, with respect to fertility and prolificacy, is not compromised. In addition, this study confirms our previous findings [5], [6] on the effectiveness of NsDU-ET, which is currently the only method available for transferring embryos deep into a uterine horn by a non-surgical procedure in pigs.

The results from Experiments 1 and 2 demonstrated that high fertilization rates, high numbers of viable embryos and low numbers of oocytes and degenerate embryos can be obtained in donors sows superovulated with 1000 IU eCG 24 h after weaning. These results are in agreement with our previous studies [6] and indicate that the superovulation treatment did not interfere with the events that control oocyte maturation, fertilization and early embryo development. It is generally assumed that superovulation treatments induce variability in the ovulatory response [20]. However, the ovulatory response variability found in the present study (CV<18%) and in our previous studies (CV = 21.4%) was much lower than previously reported values of approximately 40% in experiments with similar superovulation treatments (800 IU-1500 IU eCG) in cyclic gilts and sows [20]–[23]. Although the reasons for these differences are unclear, these studies used different lines and breeds, which can exhibit widely variable superovulatory responses [7], possibly explaining such discrepancies.

To develop a practical and effective protocol for embryo transportation, we selected two culture media and a specific stage of embryo development. We chose two culture media that did not require CO_2_ gassing to avoid additional requirements during the transport of embryos in a liquid stage: the defined TL-PVA medium, which was used in the collection, handling and washing of porcine embryos [6], [24], , and a modification of the widely used semi-defined NCSU-23 embryo culture medium supplemented with BSA [19], [26], [27], in which bicarbonate was partially replaced by HEPES (NCSU-BSA).

Because the in vitro culture conditions have a detrimental effect on embryo quality [8]-[11], [28], we decided to use the shortest culture period compatible with the commercial shipment of embryos. For this reason, we chose embryos at the morula stage for short-term storage. With these embryos, there should be at least a 24 h period before they develop to the hatching or hatched blastocyst stage. Embryos at these stages have an un-intact ZP, and for hygienic reasons, are not appropriate for transporting [29]. A period of 24 h between the collection and transfer should be sufficient for the regional, national and even international transportation of the embryos to their recipients. In addition, although fresh morulae have been successfully used in combination with NsDU-ET, the transfer of embryos at the blastocyst stage is preferred because it allows the use of recipients with a higher asynchrony degree [6]. Therefore, blastocysts derived from cultured morulae might be ideal for practical ET.

Our results clearly demonstrate that the semi-defined medium was more efficient for the short-term culture of morulae at 25°C than defined medium. Culture in the semi-defined medium at 25°C resulted in a lower proportion of degenerated embryos (17.4%) compared with that obtained in the defined medium (54.2%), although the proportion was higher in comparison with the controls (0%). By contrast, most embryos cultured at 37°C survived after 24 h of culture (>95%), with no differences compared to the controls, regardless of the medium. Although several reports have been published on the short-term culture of mammalian embryos [30]–, all used hypothermic storage temperatures (0–10°C), thereby limiting a direct comparison with our results. To our knowledge, the only previous study evaluating the effects of different temperatures and media during the short-term culture of pig embryos is that by Rubio Pomar et al [13]. These authors obtained high embryo survival rates after 24 h culture at 25°C in all of the media used. The apparent discrepancy with our results could be related to the different composition of the culture media used. Because the objective of our study was to define a practical protocol to culture pig embryos and not to evaluate culture conditions, the effects of the different medium components on embryo viability was not addressed. However, we could speculate that the presence of BSA in the culture medium could play a principal role in preventing embryo damage during culture at 25°C. Two principal facts support this assumption. First, BSA is a macromolecule widely used in embryo culture media for supporting pig embryo development in vitro [37]–[38], and second, in our study and in that by Rubio Pomar et al [13], high embryo survival rates were obtained at 25°C using media supplemented with BSA. Although the protective effects of BSA might improve the viability of pig embryos during culture at 25°C, further experiments are needed to confirm this hypothesis. The presence of BSA in the culture media may also explain the discordance between our results and those of Rubio Pomar et al [13], who reported no influence of culture temperature on embryo viability. In our study, the number of degenerated embryos decreased dramatically from 54.2% to 5% with temperature increases from 25°C to 37°C when the embryos were cultured in the defined medium. However, although significant, the decrease was more moderate (from 17.4% to 5%) when the medium containing BSA was used for the culture. This result is consistent with the results of Rubio Pomar et al [13], who used a culture medium supplemented with BSA. Somehow our results demonstrate the importance of the culture temperature on embryo viability, at least with defined culture medium.

In our study, a delay in embryo development after 24 h of culture was observed in all experimental groups compared with the controls. The delay was more marked at 25°C than at 37°C, and in both cases, it was not related to the culture medium. Most embryos were in the early and full blastocyst stages when cultured at 25°C and 37°C, respectively, whereas the expanded and hatched blastocyst stages were the most common stages observed in the control embryos. The differences in the embryo development among groups influenced the outer diameter, ZP thickness and number of cells of the embryos. These results are consistent with those previously reported, in which the embryo diameter and total number of cells increased significantly in embryos cultured at 38°C when compared with those cultured at 25°C, regardless of the culture medium [13]. A similar delay in embryo development has been reported in other species [39], [40].

Interestingly, at 25°C there was progression in the development stage (from morula to early blastocyst stage) without increasing size and cell numbers. Moreover, the number of cells of embryos of a similar developmental stage and size was lower in embryos cultured at 25°C than in those cultured at 37°C. These data indicate that morulae cultured at 25°C are able to develop during culture but that cellular proliferation is severely reduced, which might be related to the retardation of metabolism, the misregulation of genes with an important role in embryo development or the alteration of other molecular events, as previously reported for embryos of other species under hypothermic conditions [34], [40]. The question of whether these embryos retain the ability to develop to term remains to be elucidated.

The main advantage of ET in pigs is the movement of genetic material with a minimal risk of disease transmission. In this context, two aspects should be considered. First, the transportation of the embryos is restricted to embryos with an intact ZP because the ZP protects the embryos from pathogens. Therefore, from a practical point of view, the use of ZP-intact embryos is mandatory. It has been reported that embryo culture at 38°C for 24 h results in a high incidence of hatching and hatched embryos [13], in contrast with the results achieved in our study where none of the embryos cultured at 37°C hatched at the end of culture. These contradictory results might be related to the initial embryo developmental stage. Whereas in our study all embryos were at the morula stage at the beginning of culture, more than 92% of the embryos used by Rubio Pomar et al [13] were early (23%) and full blastocysts (69%). It seems clear that some of the early blastocysts and a high proportion of full blastocysts hatched at the end of culture, even taking into account the developmental delay of these embryos during culture. Hence, embryos at the morula stage should be preferred for short-term embryo culture because they retain 24 of autonomy and are less likely to hatch during short-term culture. Second, the absence of animal components in the culture media is also fundamental for sanitary and safe national and international trade of embryos. The use of serum or serum components carries with it a risk of disease transmission [15], and this fact is an important limitation for embryo movement. The use of chemically defined media for the handling and culture of the embryos should improve the reliability and reproducibility of results, reducing the risk of disease transmission. For these reasons, in experiment 2, we selected the defined TL-HEPES medium to evaluate the effects of embryo culture for 24 h at 37°C on farrowing rates and prolificacy after non-surgical deep intrauterine ET. The results from this experiment strongly confirm our findings from experiment 1 on the survival and developmental stage of the embryos cultured under these conditions. The results also demonstrated that, despite the above-mentioned embryo developmental delay, blastocysts derived from cultured morula maintained their potential to develop to term in the same way as fresh-collected blastocysts. This finding is in agreement with previous reports that indicated that the delay in the development of mouse morulae due to culture did not compromise the in vivo viability after transfer [40].

In the present study, we used a unique technique for deep-uterine non-surgical embryo transfer in pigs that was developed several years ago in our laboratory [5]. The farrowing rates were above 90%, with more than 9 piglets born. These results are comparable to our previous data reported using the same ET procedure, with a similar number of embryos per transfer [6], which confirms the effectiveness of NsDU-ET technology.

In conclusion, the results of this study indicated that TL-PVA provided a chemically defined medium capable of maintaining a high in vitro viability of porcine morula cultured at 37°C for 24 h. More than 95% of the embryos cultured under these conditions progressed to the unhatched blastocyst stage during culture, which is the most appropriate stage for NsDU-ET. Although culture caused certain embryo developmental delays, the resulting blastocysts retained their potential to develop to term in the same way as uncultured blastocysts. The excellent reproductive performance of the recipients after the NsDU transfer of embryos cultured for 24 h in a chemically defined medium reported in this study opens new possibilities for the sanitary and safe national and international trade of porcine embryos and the practical application of ET in pigs under field conditions.
